# Homologue distribution patterns of 2,3,7,8-chloro-substituted PCDD/F in Bavarian soils

**DOI:** 10.1186/s12302-017-0126-9

**Published:** 2017-11-09

**Authors:** Raimund Prinz

**Affiliations:** Bavarian Environment Agency, Hof, Germany

**Keywords:** PCDD/F-homologues, Soil samples, Forest organic layers, Top soil layers, Ubiquitous emission

## Abstract

**Background:**

Soil-background values of PCDD/F concentrations are usually conveniently displayed as toxic equivalent (TEq), being a bulk parameter of all relevant 7 PCDD- and 10 PCDF-congeneres, chloro-subsidized at the 2nd, 3rd, 7th and 8th carbon atom. Data here are ample, not so survey on congenere/homologue patterns occurring in soils. The sufficient number of samples taken within this analysis allowed first a well-grounded evaluation.

**Results:**

OCDD proved to be the dominant congenere in all samples (forest and agriculture), however, in considerably different concentrations. As expected, highest level was detected in forest organic layers, followed by forest top soils, cropland- and grassland top soils. Although highest in concentration, OCDD only contributes to 0.17% (both forest organic- and top soil layer), respectively, 0.4% (cropland) and 0.3% (grassland) to TEq. The influence of lower chlorinated homologues on TEq is strongest in forest top soils (72%) followed by 67% (forest organic layers), cropland top soil (63%) and grassland (61%). Although all homologue patterns (forest and agriculture) give a fairly similar picture, a test of significance (*χ* ^2^) proved a different population of forest samples.

**Conclusions:**

The resemblance of the homologue patterns throughout all utilization suggests that agent levels are explained mainly by diffuse atmospheric depositions rather than by specific land use input.

## Introduction

**Fig. 1:**
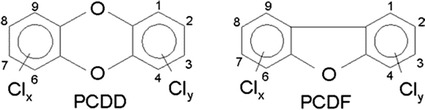
Structure of PCDD and PCDF (Taken from Frauenhofer IVV)

Polychlorinated dibenzo-*p*-dioxins and dibenzofurans are amongst the most persistent and toxic pollutants known in soils (e.g. [1, 2]. Significant and toxically hazardous are 7 dibenzodioxin- and 10 dibenzofuran-congeners, being subsidized at the 2nd, 3rd, 7th and 8th carbon atom via chlorine. 2,3,7,8-TCDD (2,3,7,8-tetra chloro dibenzodioxin) also known as a major contaminant in Agent Orange (a herbicide used during the Vietnam War) [3] is considered to be the most harmful congener. It is, therefore, provided with factor 1 within calculation of the bulk parameter “Toxic Equivalent” (TEq). All other congeners adding to TEq vary within orders of magnitude (0.1 up to 0.0001) [4] (Fig. 1).

TEq as a bulk parameter, however, does not allow detailed insight onto single congeners or homologues of PCDD/F which in return can give valuable information on the origin of the PCDD/F emission, as PCDD/F usually is a side-product of organic synthesis and burning organic material. The congener-/homologue contents are, therefore, considered to be fingerprints.

In general, higher chlorinated homologues are more stable and, therefore, longer lasting and persistent in soils [5]. Oxidative biodegradation in soils is executed mainly by microorganisms and some fungi favoring humid, organic and pH-neutral soils. The less chlorine is bound to the benzene rings the more rapid degradation follows. Degradation of higher chlorinated homologues seems to occur favourably under anaerobic conditions. Therefore, the abundance of higher chlorinated homologues outnumbers that of the lower chlorinated homologues. This matches quite well with research done on atmospheric samples carried out by Castro-Jiménez et al. [6].

To get a better picture on the distributive situation of lcH and hcH in Bavarian forest- and agricultural soils, it seemed worthwhile to compare lcH and hcH via a dimensionless quotient (*Q*_H_) (cf. Eq. 1).

1 $$Q_{\rm H} = \frac{{\sum {{\rm lcH}} }}{{\sum {{\rm hcH}} }}\quad {\rm with}\; Q_{\rm H} < 1$$

### Hypothesis

The more similar *Q*_H_ of forest- and agricultural samples derived from top soil layers are the less anthropogenic influence can be postulated and accordingly the stronger ubiquitous impact appears to be. As hcH is very much more abundant than lcH, *Q*_H_ will always be < 1.

Besides the comparison between forest- and agricultural top soil samples, it also appeared to be worthwhile taking a closer look at possible differences between crop- and grassland top soil samples. Due to alleged higher anthropogenic impact, cropland samples could presumably be slightly higher in concentration than those deriving from grassland.

## Materials and methods

The ∑PCDD/F concentrations are displayed as toxic equivalent [TEq WHO 1998 in Van den Berg et al. [7]] in [ng kg^−1^] in dry matter (DM) of fine soil and the background values both for TEq and the homologues are defined as 90% percentile of the outlier eliminated sample collective. Outliers are considered to be concentrations exceeding the 1.5 interquartile deviations. Values below detection limits are computed as 0.00 (lower bound). Homologue concentrations are not converted to TEq and are shown as detected in ng kg^−1^ DM.

The following 7 PCDD- and 10 PCDF-congeners—all chloro-subsidized at the 2nd, 3rd, 7th and 8th carbon atom—were analysed:

**Table:** 

PCDD	PCDF
2378-TetraCDD	2378-TetraCDF
12378-PentaCDD	12378-PentaCDF
123478-HexaCDD	23478-PentaCDF
123678-HexaCDD	123478-HexaCDF
123789-HexaCDD	123678-HexaCDF
1234678-HeptaCDD	123789-HexaCDF
12346789-OctaCDD	234678-HexaCDF
	1234678-HeptaCDF
	1234789-HeptaCDF
	12346789-OctaCDF

Lower chlorinated homologues are considered to be the tetra- and penta-chlorinated PCDD/F, whereas the higher chlorinated homologues comprise the hexa-, hepta- and octa chlorinated PCDD/F.

**Fig. 2:**
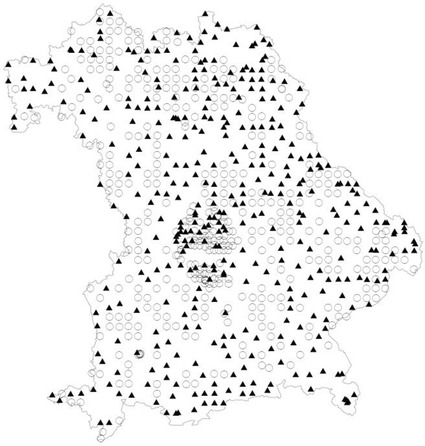
Sampling sites (circle: agriculture; triangle: forest )

Samples were all taken only in rural regions to excerpt background values. All samples derive from soil profiles burrowed at least 1 m in depth. Soil horizons were labelled according to KA4 [8]. Sampling method was executed horizon wise according to LABO [9]. Top soil horizons are hence all horizons up to max. of 30 cm depth and/or all A-Horizons. Organic layers were separated in the field by soilscientists using a steel framework 20 × 20 × 10 cm. Figure 2 displays the sample sites (forest, crop- and grassland). Analysis is executed on dry matter (105 °C) (DIN ISO 11465:12.96 [10]). The mineral compounds were conducted on fine soil (< 2000 µm grain size) by application of DIN 19683-2:04.97 [11]. Organic layers were dried and then ground to particle size of 60 µm bevor analysed (DIN ISO 11464 [12]).

*Detection method* Extraction via toluol (20 h, Soxhlet); chromatographic purification via silica gel/sulfuric acid, quantification analysis: GC-HRMS (DIN38414-24:04.98 [13]).

## Results

### Homologues

The examination of homologues within different land use (forest versus agriculture) should show a more or less similar homologue pattern, unless anthropogenic impact has an overwhelming effect upon the samples taken from agricultural sites. Forest samples, especially organic layers, usually show higher concentrations in comparison to agricultural samples as forest sites—foremost coniferous forests—function as a kind of filter (e.g. [14]). And persistant organic pollutants (POPs) are strongly lipophilic and, therefore, predominantly attached to the organic matter.

A comparison between grass- and cropland aims to identify feasible anthropogenic effects. Cropland is usually subject to higher anthropogenic input.

### Forest organic layers

Table 1 displays descriptive statistical results of the organic layers. Homologues of (tetra), (penta), (hexa), (hepta) and (octa) chlorinated dibenzodioxin (CDD) and dibenzofuran (CDF) are displayed.

**Table 1:** Descriptive statistical results of PCDD/F-homologues [ng kg^−1^ DM] in organic layers of Bavarian forest soils

	TCDD	PeCDD	HxCDD	HpCDD	OCDD	TCDF	PeCDF	HxCDF	HpCDF	OCDF	TEq
*n*	313	315	314	311	314	311	312	312	311	310	312
*O* (*n*)	4	2	3	6	3	6	5	5	6	7	5
Min	0.00	0.00	0.00	0.00	0.00	0.00	0.00	0.00	0.00	0.00	0.00
Max	2.3	14	53	200	1100	46	82	112	191	185	57
MV	0.59	3.6	15	61	314	14	21	33	50	44	18
Med	0.51	3.0	13	52	250	13	18	28	41	33	15
90	*1.2*	*6.8*	*27*	*104*	*580*	*25*	*39*	*61*	*94*	*92*	*33*

*O* (*n*), number of outliers; MV, mean value; Med, median; 90, 90% percentile

**Fig. 3:**
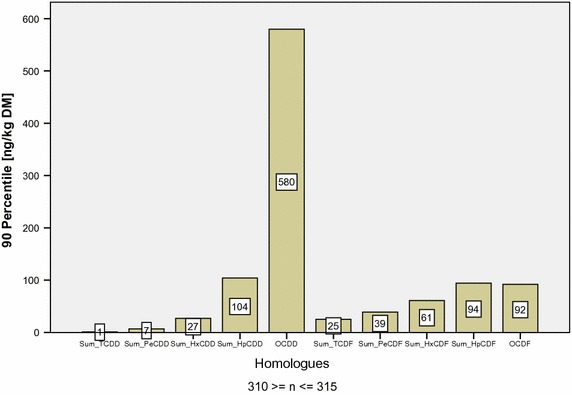
Homologue pattern of 2,3,7,8-chloro subsidized PCDD/F in organic layers of Bavarian forest soils (outlier eliminated)

The homologue distribution of organic layers is visualized in Fig. 3 using the 90% percentile.

As Fig. 3 displays, OCDD concentrations clearly outnumber all other homologues. However, multiplied with the WHO-TEq-Factor of 0.0001, OCDD only contributes to 0.17% of the 90% percentile of TEq (33.3 ng kg^−1^ DM). In contrast, 67% of TEq is generated by the sum of lcH DD/F-homologues.

The calculated* Q*_H_ for organic layers is 0.08 as the percentage of lcH to hcH bears a ratio of 7:93.

**Fig. 4:**
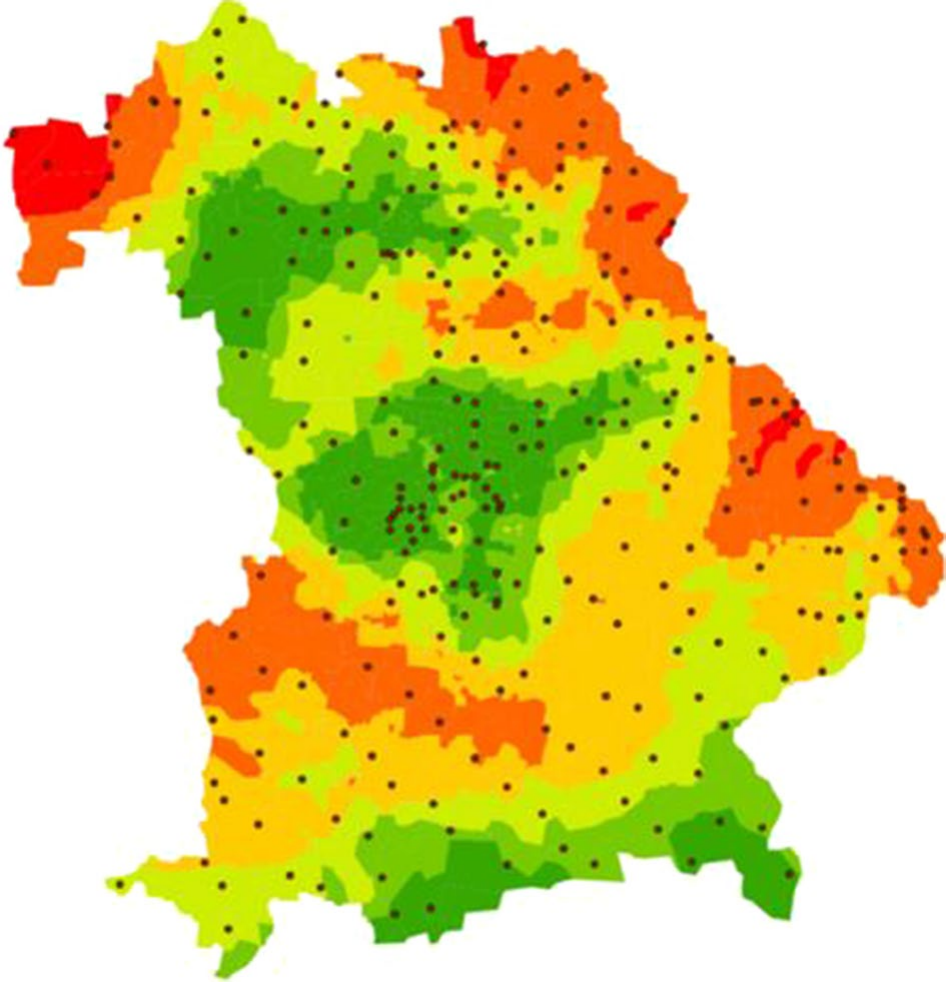
Distribution of 6 differing subspaces of PCDD/F-concentrations in organic layers

**Fig. 5:**
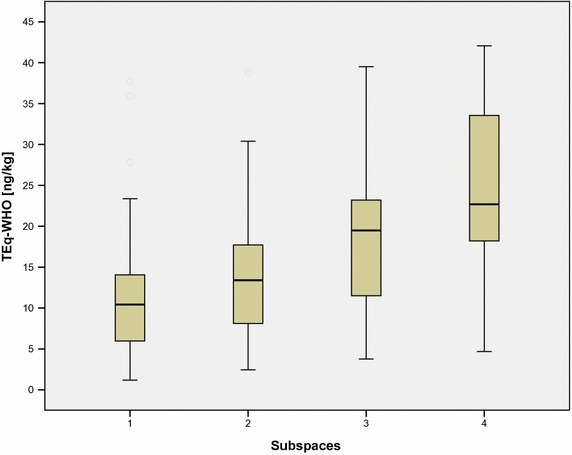
Box–Whisker plot of the aggregated PCDD/F subspaces

Within forest sites, Hangen and Prinz (Bayerisches Landesamt für Umwelt [15]) could, however, identify regions with diverging concentrations of PCDD/F in organic—as well as in top soil layers (cf. Figs 4 and 5).

Higher concentrations detected in NE-Bavaria are most probably due to brown coal emissions caused by the close by Czech coal-power plants in Sokolov. Those in NW Bavaria are assumed to be caused by the nearby industrial region of Frankfurt [16].

A median comparison of some selected congeners within the subspaces (Sub) 1–4 (cf. Fig. 5) is to be seen in Table 2.

**Table 2:** Median comparison of selected congeners within subspaces

	Sub 1 82 ≥ n ≤ 89	Sub 2 46 ≥ n ≤ 47	Sub 3 76 ≥ n ≤ 78	Sub 4 78 ≥ n ≤ 79
2,3,7,8 TCDD	9.4	11.0	15.3	17.0
2,3,7,8 TCDF	0.3	0.43	0.63	0.7
1,2,3,6,7,8 HxCD	4.4	5.9	6,5	9.6
1,2,3,6,7,8 HxCF	6.0	7.3	9.6	14.0
OCDD	160	220	263	370
OCDF	22.0	30.0	33.3	62
TEq	10.5	12.9	19.4	22.7

Looking at the data of Table 1 and comparing the median of TEq (15 ng kg^−1^) for the overall population (*n* = 312) with the median of the subspaces varying from 10.5 to 22.7, the overall median of 15 ng kg^−1^ seems to be quite well in the middle implicating a more or less homogenies distribution and hence proposing the applicability of forests representing background values.

### Forest top soil layers

**Fig. 6:**
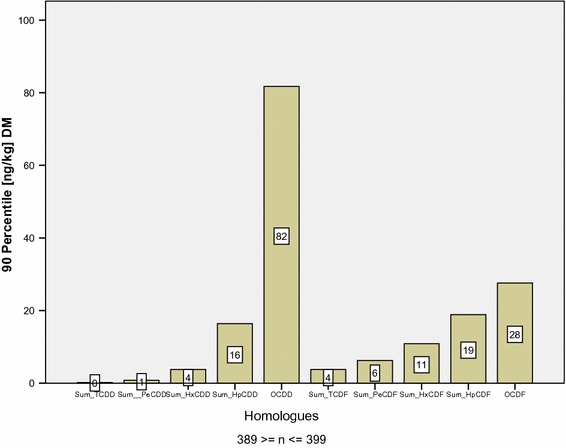
Homologue pattern of 2,3,7,8-chlorine subsidized PCDD/F in Bavarian forest top soil layers (outlier eliminated)

As expected top mineral layers of forest soils show a different picture in quantity. Organic pollutants such as PCDD/F are lipophilic and are, therefore, highly attached to soil organic matter [17] on the one hand; on the other hand, also specific weight in mineral top soils is very much higher. Hence, mass-related values in mineral top soil layers are usually lower in concentration (cf. Table 3 and Fig. 6).

**Table 3:** Descriptive statistical results of PCDD/F-homologues [ng kg^−1^ DM] in Bavarian forest top soil layers

	TCDD	PeCDD	HxCDD	HpCDD	OCDD	TCDF	PeCDF	HxCDF	HpCDF	OCDF	TEq
*n*	399	393	396	391	389	386	392	395	399	398	394
*O* (*n*)	10	16	13	18	20	23	17	14	10	11	15
Min	0.00	0.00	0.00	0.53	2.50	0.00	0.00	0.00	0.00	0.00	0.04
Max	0.33	1.6	7.7	29	145	6.8	11	21	38	48	8.5
MV	0.07	0.4	2.0	8.1	42	1.8	3.1	5.0	10	14	2.6
Med	0.05	0.3	1.6	6.1	31	1.2	2.4	3.8	8.2	12	2.1
90	*0.2*	*0.8*	*3.8*	*16*	*82*	*3.7*	*6.4*	*11*	*19*	*28*	*4.8*

*O* (*n*), number of outliers; MV, mean value; Med, median; 90, 90% percentile

As can be shown in Fig. 6 OCDD values are also dominant in the mineral soil components but by a far lower margin. In contrast to the organic soil layer though, OCDD- and OCDF rates in mineral top soils are very much closer. OCDD values in organic layers are five times higher than those of OCDF, whereas in mineral layers the factor only reaches up to three.

Just as in organic layers OCDD contributes merely 0.17 to the 90% percentile of TEq (4.8 ng kg^−1^ DM). TCDD/F and PeCDD/F in contrast account for 72% of TEq.

The calculated *Q*_H_ for mineral forest top soil layers is 0.07 as the percentage of lcH to hcH bears a ratio of 6:94.

### Agricultural top soil layers

#### Cropland

Due to anthropogenic influences, agricultural soils differ not only in their pedogenetic phenotype but also in agent content in comparison to forest soils. On the one hand, permanent crop yield will subduct nutrients as well as pollutants; on the other hand, there is an extra input by adding farm- and mineral fertilisers. Farm fertiliser will boost humus content, which of course is beneficial in many ways. Mineral fertilisers in contrast often contain harmful pollutants such as Uranium or Cadmium. Sewage sludge occasionally even contains traces of endocrine disruptors and other harmful organic substances or their metabolites which finally led to the ban of application of sewage sludge as a fertiliser in Germany within the next 10 years. In 2014, a total of 283,000 tons of sewage sludge incurred in Bavaria; 22.4% were deployed on agricultural soils.^1^

Mineral top soil samples from cropland and grassland alike show dominant OCDD concentrations with the highest ratio of influence on TEq by cropland with 0.4%. OCDD concentrations in grassland only account for 0.3% on total TEq.

The calculated *Q*_H_ for cropland top soil layers is 0.03. Tetra- and penta-chlorinated DD and DF account for 63% of TEq (Table 4).

**Table 4:** Descriptive statistical results of PCDD/F-homologues [ng kg^−1^ DM] in Bavarian cropland top soil layers

	TCDD	PeCDD	HxCDD	HpCDD	OCDD	TCDF	PeCDF	HxCDF	HpCDF	OCDF	TEq
*n*	217	223	222	221	215	223	221	222	220	218	217
*O* (*n*)	17	11	12	13	19	11	13	12	14	16	17
Min	0.00	0.00	0.00	0.00	0.00	0.00	0.00	0.00	0.00	0.00	0.00
Max	0.1	0.5	2.4	13	71	1.5	2.0	4.7	6.5	12	1.7
MV	0.02	0.1	0.7	4.1	23	0.5	0.7	1.4	2.4	3.6	0.7
Med	0.00	0.1	0.6	3.4	18	0.4	0.7	1.3	2.3	3.2	0.6
90	*0.04*	*0.2*	*1.1*	*6.8*	*43*	*0.7*	*1.2*	*2.1*	*3.5*	*5.8*	*1.0*

*O* (*n*), number of outliers; MV, mean value; Med, median; 90, 90% percentile

#### Grassland

Data in Table 5 display a similar homologue pattern to cropland. The abundance of OCDD (factor 5 higher than OCDF) also proves to be characteristical in grassland samples (cf. Fig. 4). The higher chlorinated homologues account for 39% of ∑TEq,* Q*_H_ with 0.03 is virtually equal to that of cropland, with a ratio of lcH to hcH (3:97) in grassland being identical to that of cropland (cf. Table 5).

**Table 5:** Descriptive statistical results of PCDD/F-homologues [ng kg^−1^ DM] in Bavarian grassland top soil layers

	TCDD	PeCDD	HxCDD	HpCDD	OCDD	TCDF	PeCDF	HxCDF	HpCDF	OCDF	TEq
*n*	215	218	210	206	206	213	207	200	191	201	194
*O* (*n*)	12	9	17	21	21	14	20	27	36	26	33
Min	0.00	0.00	0.00	0.82	3.0	0.08	0.00	0.20	0.40	0.29	0.12
Max	0.1	0.6	2.5	13	72	1.5	2.2	4.7	7.1	12	1.8
MV	0.02	0.14	0.91	4.9	24	0.51	0.89	1.7	2.9	4.3	0.76
Med	0.01	0.1	0.8	4.3	21	0.41	0.79	1.6	2.8	3.8	0.71
90	*0.04*	*0.2*	*1.3*	*7.4*	*38*	*0.7*	*1.4*	*2.6*	*4.8*	*6.9*	*1.2*

*O* (*n*), number of outliers; MV, mean value; Med, median; 90, 90% percentile

**Fig. 7:**
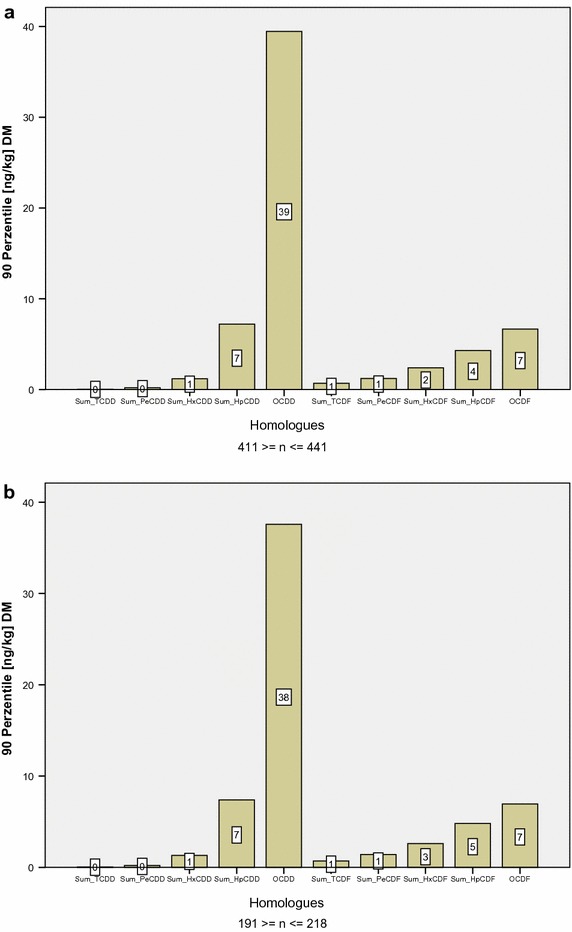
**a** Homologue pattern of 2,3,7,8-chlorine subsidized PCDD/F in Bavarian cropland top soil layers (outlier eliminated). **b** Homologue pattern of 2,3,7,8-chlorine subsidized PCDD/F in Bavarian grassland top soil layers (outlier eliminated)

Table 6 finally displays the homologue quotient and—pattern of the lower—and higher chlorinated homologues from forest and agricultural samples. The closest rate between lcH and hcH is detected in organic layers followed by forest top soil samples. Agricultural soil samples are within identical range (Fig. 7). The influence of lcH upon TEq is highest in forest samples, thus, allowing the following sequence: *Q*_H_-Organic layer (0.08) > *Q*_H_-Forest top soil (0.07) > *Q*_H_-Grassland top soil (0.03) ≈ *Q*_H_-Cropland top soil (0.03).

**Table 6:** Comparison between forest and agricultural samples

	Forest				Agriculture			
	Organic layer		Top soil		Cropland top soil		Grassland top soil	
	lcH	hcH	lcH	hcH	lcH	hcH	lcH	hcH
*H*_p_	7	93	6	94	3	97	3	97
*H*_P_-TEq	67	33	72	28	63	37	61	39
*Q*_H_	*0.075*		*0.068*		*0.031*		*0.032*	

*Q*_H_, homologue quotient of lcH to hcH [dimensionless]; *H*_p_, homologue pattern of lcH to hcH [%]; *H*_p_-TEq, homologue pattern of lcH and hcH in terms of TEq [%]

## Discussion

In order to get an idea whether the specific forest-, crop-, and grassland top soil samples derive from identical or diverse populations a test of significance *χ* ^2^ (Chi square) was executed. Both clusters, the lcH as well as the hcH, were examined with regard to their origin of land use.

As shown in Table 7 lcH- and hcH-data deriving from forest samples prove to be a different population on a probability of *α* = 0.05 in comparison to those deriving either from crop- or grassland.

**Table 7:** Test of significance (*χ* ^2^) of lcH and hcH taken from forest (F)-, crop (C)- and grassland (G) mineral top soil samples

	lcH			hcH		
	F versus C	F versus G	C versus G	F versus C	F versus G	C versus G
H	H_1_	H_1_	H_1_ (H_0_)*	H_1_	H_1_	H_1_ (H_0_)*

H: Hypothesis (*α* = 0.05); H_0_: Samples belonging to same population; H_1_: Samples deriving from diverse population

*cf. text

### H_1_ (H_0_)*

Crop- and grassland lcH-/hcH-data are to be interpreted with great care, as the *χ* ^2^ test value of *χ* ^2^ = 2.706 for lcH is barely exceeded (^*χ* ^2^ = 2.765). Difference between the hcH of crop- and grassland data seems to be greater (^*χ* ^2^ = 30.15) yet is still to be considered faint in comparison to the other combinations which reign within multitudes of hundreds. A Kruskal–Wallis (*H* test) verifies these results and conclusions.

The only conclusion which with reasonable certainty can be drawn out of these statistical tests is that forest samples obviously differ quite clearly from samples taken from agricultural sites. If, as presumed, PCDD/F-emission originates from ubiquitous sources then the reason that forest samples differ from agricultural ones can be assumed in the manner forest sites filter atmospheric pollutants and/or accumulation rates are higher in forest soils due to the lack of dilution via anthropogenic interactions such as, e.g. ploughing. Specific land use practice such as ploughing, harrowing and liming (in agricultural sites) may promote rapid microbiological breakdown of the lower chlorinated dioxins and—furans in comparison to forest top soil contents. Also pH values and organic carbon contents differ considerably in forest soils in comparison to soils under agricultural sites: examined forest organic layers reveal mean values of pH 3.7, their top soil layers reveal mean values of pH 3.6, while cropland top soils show pH values of 6.3 and grassland top soils 5.8. Both organic carbon and pH are significant parameters relating to PCDD/F accumulation or breakdown [18]. These facts may also account for the diverse population portrayed in Table 7.

### Data of the German UBA

**Fig. 8:**
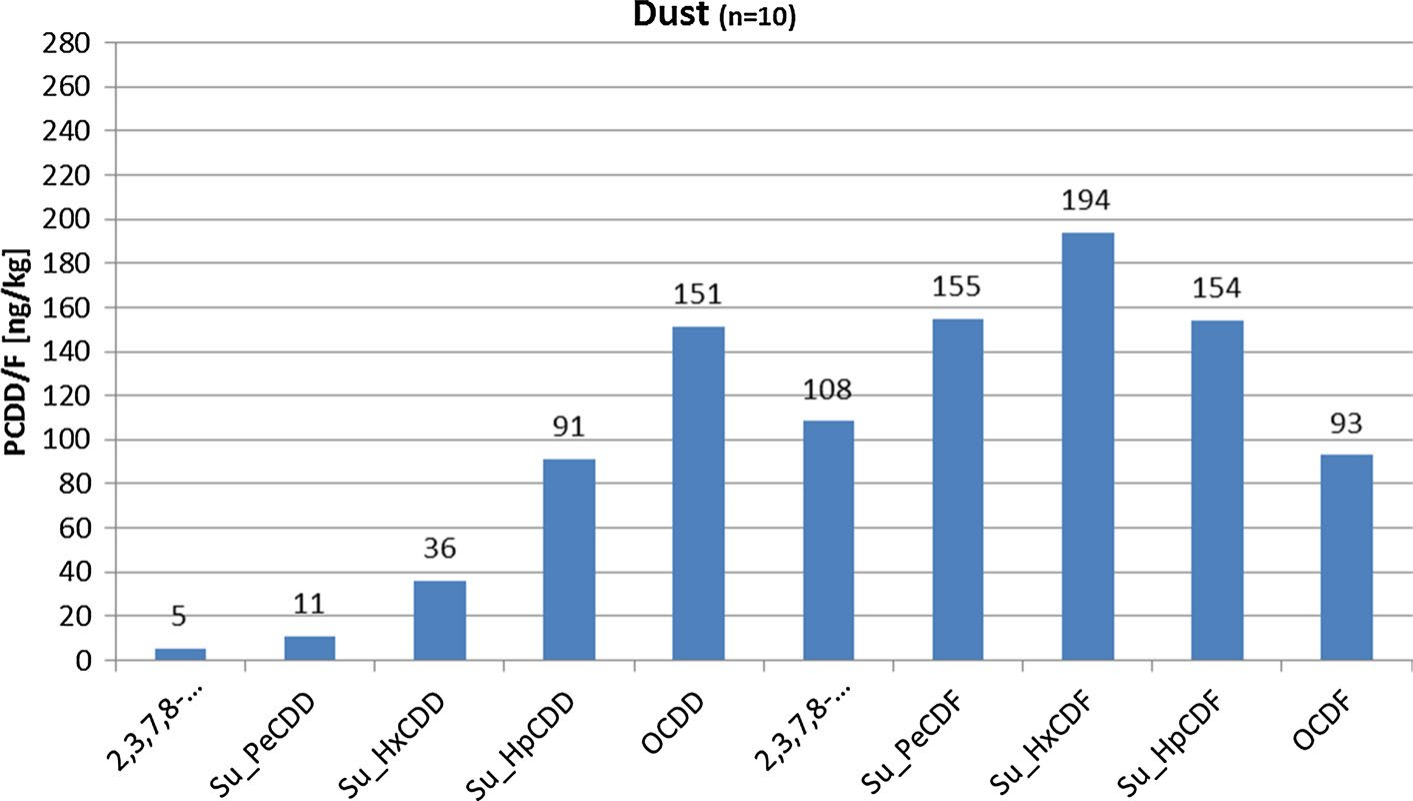
Homologue pattern of dust (UBA) (Data taken from [19])

Comparison with data of the German Environmental Protection Agency (Umwelt Bundesamt (UBA)) indicates that emission-related homologues from, e.g. dust show considerably different patterns (cf. Fig. 8) and do not match with patters related to soil probes taken from rural areas. Soil background values have their peak at OCCD, whereas dust has peaks at higher chlorinated PCDF’s. Dust emissions therefore do not—as possibly presumed—dominate PCDD/F patterns in rural soils.

Samples deriving from wood, biodiesel, solvents and synthetic material analysed by the UBA also all show no resemblance to the homologue patterns in soils.

**Fig. 9:**
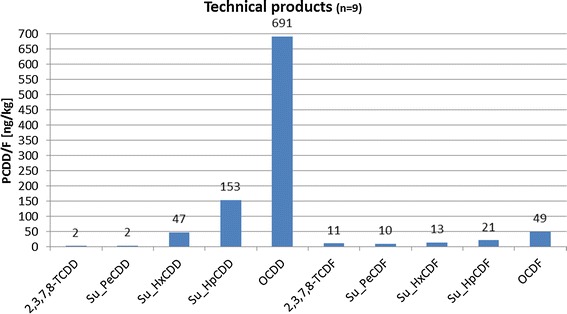
Homologue pattern of technical products (UBA) (Data taken from [19])

PCDD/F-emissions originating from technical products (cf. Fig. 9) in contrast seem to match quite well with the homologue pattern of soil samples. Both homologue patterns peak at OCDD with raised values at HxCDD/F, HpCDD/F and OCDF. This seems to indicate that PCDD/F values of soil samples deriving from rural areas may nowadays be predominately influenced by emissions originating from technical products.

## Conclusions

1. Data confirm predominance of OCDD in all samples (forest, crop- and grassland).
2. *Q*_H_ in forest soils is highest, hence concentrations of lower chlorinated homologues are in comparison to agricultural sites also highest and, therefore, influence TEq strongest.
3. TEq under forest use is, therefore, mainly determined by lcH (72%), while crop- and grassland sites are “merely” influenced by 63% resp. 61%.
4. Samples deriving from forest sites are statistically part of a different population (*χ* ^2^ (Chi square) test of significance and *H* test) because of quantity, the patterns, however, are similar.
5. Calculated *Q*_H_ and relation of lcH to hcH in crop- and grassland soils are virtually of one and the same magnitude.
6. Similarity of the homologue patterns of cropland on the one hand and grassland on the other suggests that there seems to be no considerable influence of land use on concentration levels of PCDD/F.
7. The similar homologue patterns of PCDD/F-concentration levels in crop- and grassland suggest that agent levels are explained mainly by diffuse, ubiquitous, atmospheric depositions rather than specific land use parameters.
8. Calculated *H*_P_ of agricultural soils may lead to the assumption that specific land use practice such as ploughing, harrowing and liming promotes rapid microbiological breakdown of the lower chlorinated dioxins and furans in comparison to forest top soil contents.
9. Homologue—comparison with data of the German Environmental Protection Agency (UBA) suggests that PCDD/F values in soils of rural areas could strongly be determined by emissions related to technical products.

## Footnotes

1
https://www.destatis.de/DE/PresseService/Presse/Pressemitteilungen/2015/12/PD15_462_32214.html;jsessionid=0FE11C0F50655FAF6DBB6E7D447C0A45.cae4.
